# Attention-Enhanced CNN-LSTM with Spatial Downscaling for Day-Ahead Photovoltaic Power Forecasting

**DOI:** 10.3390/s26020593

**Published:** 2026-01-15

**Authors:** Feiyu Peng, Xiafei Tang, Maner Xiao

**Affiliations:** School of Electrical and Information Engineering, Changsha University of Science and Technology, Changsha 410114, China; 202304220319@csust.edu.cn (F.P.); manerxiao@stu.csust.edu.cn (M.X.)

**Keywords:** photovoltaic power forecasting, CNN-LSTM, attention mechanism, numerical weather prediction, spatial downscaling, multi-source data fusion, deep learning

## Abstract

Accurate day-ahead photovoltaic (PV) power forecasting is essential for secure operation and scheduling in power systems with high PV penetration, yet its performance is often constrained by the coarse spatial resolution of operational numerical weather prediction (NWP) products at the plant scale. To address this issue, this paper proposes an attention-enhanced CNN–LSTM forecasting framework integrated with a spatial downscaling strategy. First, seasonal and diurnal characteristics of PV generation are analyzed based on theoretical irradiance and historical power measurements. A CNN–LSTM network with a channel-wise attention mechanism is then employed to capture temporal dependencies, while a composite loss function is adopted to improve robustness. We fuse multi-source meteorological variables from NWP outputs with an attention-based module. We also introduce a multi-site XGBoost downscaling model. This model refines plant-level meteorological inputs. We evaluate the framework on multi-site PV data from representative seasons. The results show lower RMSE and higher correlation than the benchmark models. The gains are larger in medium power ranges. These findings suggest that spatially refined NWP inputs improve day-ahead PV forecasting. They also show that attention-enhanced deep learning makes the forecasts more reliable. Quantitatively, the downscaled meteorological variables consistently achieve lower normalized MAE and normalized RMSE than the raw NWP fields, with irradiance-related errors reduced by about 40% to 55%. For day-ahead PV forecasting, using downscaled NWP inputs reduces RMSE from 0.0328 to 0.0184 and MAE from 0.0194 to 0.0112, while increasing the Pearson correlation to 0.995 and the CR to 98.1%.

## 1. Introduction

The rapid growth of grid-connected photovoltaic (PV) generation is driving the decarbonization of modern power systems [1,2]. However, PV output is inherently intermittent and nonlinear because irradiance and local weather vary rapidly, which complicates power balancing, frequency stability, and economic dispatch, especially in low-inertia grids with high renewable penetration [3]. Accurate day-ahead PV power forecasting is therefore critical for secure operation, reserve scheduling, market bidding, and flexibility planning [4]. Recent reviews show that forecast accuracy affects both the economic value of renewable assets and the overall operating cost of power systems [4,5]. They also report a growing reliance on numerical weather prediction (NWP) products and advanced machine learning and deep learning models across forecasting horizons and spatial scales [6]. In addition, the research focus has shifted from single-plant forecasting toward regional and multi-site frameworks [7].

From a modelling perspective, PV power forecasting approaches can be broadly categorized into physical, statistical, and data-driven models [2]. Physical or physics-informed methods rely on radiative transfer equations, PV performance models, and detailed NWP inputs, and are capable of providing physically interpretable forecasts at both plant and regional scales [8,9]. Their performance, however, depends strongly on the availability of accurate NWP fields, high-quality system configuration data, and carefully calibrated parameter sets, which are often unavailable in operational environments or difficult to maintain as plants age. Statistical and shallow ML models—including autoregressive integrated moving average (ARIMA), support vector regression, random forests, and gradient boosting—have been widely adopted owing to their simplicity and relatively low computational cost [10,11]. Nevertheless, these models generally struggle to capture the complex nonlinear spatio-temporal dependencies inherent in PV power time series, especially under highly variable weather conditions and in systems with strong diurnal and seasonal non-stationarity [3,9].

With the proliferation of high-frequency supervisory control and data acquisition (SCADA) measurements and large-scale meteorological datasets, DL-based methods have become mainstream for short-term and day-ahead PV power forecasting [12]. Recurrent neural network (RNN) architectures, particularly long short-term memory (LSTM) networks, have demonstrated a strong ability to model long-range temporal dependencies in non-stationary PV power series and irradiance sequences [13,14]. Several studies have shown that LSTM-based models can outperform classical statistical and shallow ML baselines in both point and interval forecasting tasks, especially when exogenous meteorological inputs such as NWP products or on-site weather data are incorporated [15]. To further exploit spatial correlations and local patterns, hybrid convolutional neural network–LSTM (CNN–LSTM) architectures have been proposed, in which CNN layers extract short-term local features while LSTM layers capture long-term temporal dynamics [16]. Such hybrids have been widely validated for multi-step PV power forecasting and irradiance prediction under diverse climates, plant configurations, and data sources, including multi-station settings with neighboring PV plants [17].

More recently, attention mechanisms and transformer-based architectures have been introduced into PV forecasting to adaptively reweight the contributions of different time steps, features, and spatial locations [18]. Attention-enhanced CNN–LSTM or bidirectional LSTM/GRU hybrids have shown that selectively focusing on key temporal segments or meteorological factors can significantly enhance forecast accuracy and robustness, particularly during cloud passages and other rapidly changing conditions [19,20]. Beyond RNN-based hybrids, transformer variants have been proposed for multi-step and day-ahead PV power forecasting, demonstrating improved representation of long-horizon dependencies and flexible handling of heterogeneous inputs [21,22,23]. In parallel, graph-based neural networks and spatio-temporal attention models have been exploited for multi-site or regional PV forecasting tasks, capturing complex spatial interaction patterns among geographically dispersed plants [7,24,25]. These developments indicate a clear trend from purely temporal models to architectures that explicitly encode both spatial and temporal structures.

Beyond network architecture design, many studies highlight the importance of numerical weather prediction (NWP) inputs and exogenous meteorological variables for improving long-horizon forecasting performance [26]. These variables typically include global horizontal irradiance, cloud cover, ambient temperature, relative humidity, and wind speed. However, operational NWP products usually have limited spatial and temporal resolution. This limitation prevents them from capturing local-scale meteorological variations that strongly influence plant-level PV power output. Consequently, systematic biases and smoothing effects in NWP fields can propagate into PV power forecasts, particularly in complex terrain or coastal regions [27,28]. Several studies have demonstrated that combining NWP information with data-driven post-processing can substantially improve both irradiance and power forecasts by correcting model biases and better exploiting local meteorological signals [29,30]. At the same time, recent reviews and methodological studies underline that the way NWP products are preprocessed, selected, and integrated into DL architectures has a non-negligible impact on forecast skill [31].

To alleviate the spatial-scale mismatch between coarse NWP grids and plant-level conditions, considerable efforts have been devoted to statistical and ML-based downscaling of meteorological variables such as solar irradiance, air temperature, and wind speed [32]. For solar radiation applications, neural-network-based downscaling and bias-correction techniques have been used to improve direct normal irradiance (DNI) and global horizontal irradiance forecasts derived from global or regional NWP models [32,33]. Physically informed or terrain-aware downscaling methods have also been proposed to account for topographic and land–sea contrast effects, providing more realistic local irradiance fields for PV performance assessment [32,34]. Beyond solar radiation, domain-informed CNNs and hybrid ML approaches have been applied to generate high-resolution wind and climate fields from coarse NWP or reanalysis data, illustrating the general potential of combining physical priors with data-driven learning for scale-bridging problems [34]. Very recently, multi-scale PV forecasting frameworks that integrate regional high-resolution NWP, physics-informed networks, and data-driven components have further highlighted the benefits of treating NWP refinement as an integral part of the forecasting pipeline [35].

For PV applications in particular, several studies have shown that carefully designed downscaling or bias-correction schemes can substantially improve NWP-driven irradiance or power forecasts at both site and regional scales [36]. Neural-network-based correction of ECMWF-based irradiance forecasts and spatially generalisable bias correction of satellite solar radiation products both point to the importance of exploiting multi-source information when bridging scale gaps [37,38]. However, many existing PV power forecasting frameworks still treat NWP inputs as fixed exogenous variables without explicit spatial refinement, or perform downscaling and power forecasting as two loosely coupled stages [39,40]. This decoupling may lead to suboptimal use of multi-source information and limit model robustness under heterogeneous or rapidly changing weather conditions, especially when only a limited number of local observations are available at the plant level [40].

In summary, the above literature reveals three key gaps. First, recent PV forecasting models, including CNN–LSTM hybrids, attention-enhanced RNNs, transformers, and graph neural networks, have shown strong performance [41,42]. However, many studies emphasize temporal feature learning or regional spatial patterns, while giving limited attention to explicitly correcting plant-level bias and spatial inconsistency in meteorological drivers. Second, several works explore regional or multi-plant forecasting with transformers, graph networks, or transfer learning [43]. These approaches often depend on coarse NWP inputs or spatially aggregated features, and they rarely use multi-site observations to refine the local meteorological fields for a specific target plant. Third, the literature on NWP downscaling and bias correction is mature in meteorology, climate science, and solar forecasting. Yet, only a limited number of studies tightly integrate these techniques with attention-enhanced deep learning for end-to-end day-ahead PV forecasting at the plant scale.

To address these gaps, this paper proposes an attention-enhanced CNN–LSTM framework with spatial downscaling and multi-source meteorological fusion for day-ahead PV power forecasting at the plant level. The main contributions are summarized as follows:

1. A leakage-free, modular two-stage pipeline is established to decouple (i) spatial representativeness correction of coarse-resolution NWP fields and (ii) temporal dependency learning for day-ahead PV power forecasting, while coupling them through a clear interface in which the downscaled site-level meteorological variables serve as the exclusive exogenous inputs to the forecaster. A strict cross-station transfer protocol is adopted such that the target station contributes no measurement pairs during downscaling training, improving practical applicability under limited target-site supervision.
2. A multi-site, multi-output spatial downscaling module is constructed using XGBoost to learn a nonlinear mapping from location/time encodings and heterogeneous NWP predictors to site-level meteorological vectors, trained only with measurement-based pairs from surrounding stations. This design enables plant-specific, high-resolution meteorological features to be generated from coarse NWP inputs, thereby reducing NWP-induced spatial bias at the target plant.
3. An attention-enhanced CNN–LSTM forecasting model is developed to jointly capture short-term local patterns and long-term temporal dependencies, and to adaptively fuse multi-source meteorological predictors (irradiance, temperature, humidity, wind, etc.) with historical PV power. Comprehensive experiments on a multi-station PV dataset across representative months and across input/model configurations validate consistent accuracy improvements over baseline architectures in terms of RMSE, MAE, and correlation.

The remainder of this paper is organized as follows. Section 2 presents the modeling and analysis of photovoltaic power output. Section 3 presents the proposed attention-enhanced CNN–LSTM architecture with spatial downscaling. The case study design and evaluation setup are detailed in Section 4. Section 5 reports and analyzes the forecasting results. Finally, Section 6 summarizes the main findings and conclusions.

## 2. Modeling and Analysis of Photovoltaic Power Output

This section presents the modeling and analysis of photovoltaic (PV) power output to support the proposed forecasting framework. The physical principles of PV generation are first summarized, followed by a theoretical formulation that relates irradiance, temperature effects, and conversion efficiency to the expected power output. Based on the theoretical results and historical measurements, the seasonal and diurnal characteristics of PV output are analyzed to reveal representative long- and short-term fluctuation patterns. The discrepancies between theoretical and measured power are then examined as an analytical reference for identifying the impact of environmental conditions (e.g., temperature, humidity, and cloudiness).

### 2.1. Theoretical Model of PV Power Output

The theoretical photovoltaic (PV) power output is determined by the total irradiance received on the tilted surface and the conversion efficiency of the PV module [44,45]. It can be expressed as

(1) $$P_{\mathrm{th}}\left(t\right)=G_{\mathrm{POA}}\left(t\right)\;\eta \left(t\right)\;A$$

where $G_{\mathrm{POA}}\left(t\right)$ is the total irradiance incident on the plane of array (W/$m^{2}$), η(t) is the PV module efficiency including temperature effects, and *A* denotes the total area of the PV array ($m^{2}$).

**(1) Irradiance model on a tilted plane:** The total irradiance on a tilted PV surface can be calculated as the sum of direct, diffuse, and ground-reflected components:

(2) $$G_{\mathrm{POA}}=G_{\mathrm{beam}}+G_{\mathrm{diffuse}}+G_{\mathrm{ground}}$$

where $G_{\mathrm{beam}}$ is the direct irradiance on the tilted surface, $G_{\mathrm{diffuse}}$ is the sky-diffuse component, and $G_{\mathrm{ground}}$ is the ground-reflected irradiance. All three components can be computed from the solar position and site geometry using standard solar-radiation models.

**(2) PV module efficiency model:** The conversion efficiency of the PV module can be described by a temperature-corrected linear model:

(3) $$\eta \left(t\right)=\eta_{\mathrm{ref}}\left[1-\beta \left(T_{\mathrm{cell}}\left(t\right)-25\right)\right]$$

where $\eta_{\mathrm{ref}}$ is the reference efficiency at 25 ∘C (typically 0.15–0.18), β is the temperature coefficient of efficiency (0.003–0.005${/}^{\circ }$C), and $T_{\mathrm{cell}}\left(t\right)$ is the cell temperature (°C). Equation (3) indicates that the module efficiency decreases approximately linearly with increasing cell temperature.

**(3) PV cell temperature model (Faiman model):** The cell temperature can be estimated using the empirical Faiman model:

(4) $$T_{\mathrm{cell}}=T_{\mathrm{air}}+\frac{G_{\mathrm{POA}}}{800}\left(20-0.5v\right)$$

where $T_{\mathrm{air}}$ is the ambient temperature (°C) and *v* is the wind speed (m/s). This model assumes that the temperature rise of the PV cell above the ambient temperature is proportional to the incident irradiance and is mitigated by wind cooling.

Combining (1)–(4), the theoretical power output can be computed under different irradiance and environmental conditions, capturing the combined effects of solar radiation, temperature, and wind. To quantify the deviation between theoretical and measured outputs, a relative deviation index is defined as

(5) $$\delta \left(t\right)=\frac{P_{\mathrm{meas}}\left(t\right)-P_{\mathrm{th}}\left(t\right)}{P_{\mathrm{th}}\left(t\right)}\times 100\%$$

where $P_{\mathrm{meas}}\left(t\right)$ is the measured output power. The deviation δ(t) captures the effects of cloud dynamics, humidity, and rapid irradiance fluctuations, and its temporal patterns provide useful guidance for feature selection and modelling choices in later sections.

### 2.2. Photovoltaic Power Generation Characteristics

**Figure 1:**
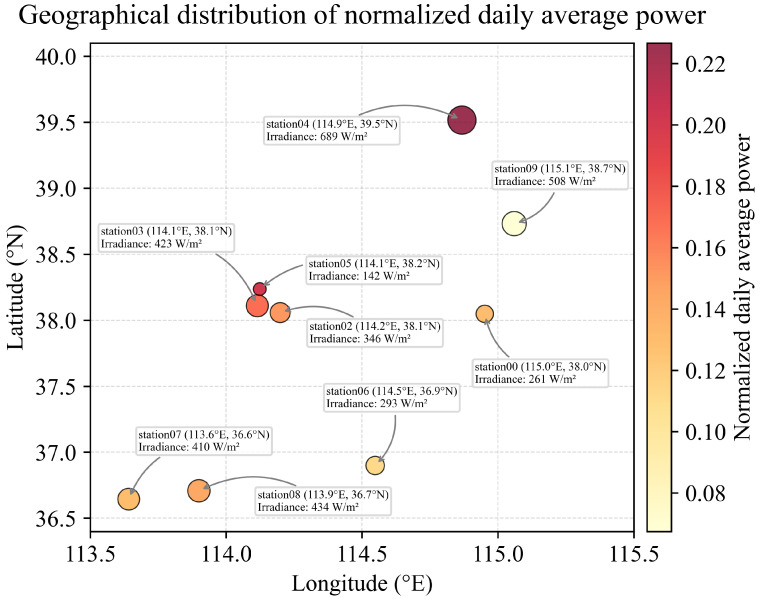
Geographical distribution and performance indicators of the ten PV power plants.

**(1) Geographical distribution and performance indicators:** Figure 1 illustrates the geographical distribution and performance indicators of the ten photovoltaic (PV) power plants. Each site is positioned according to its latitude and longitude, with the marker size representing the annual average plane-of-array irradiance and the color scale denoting the normalized daily average power output. All sites are located in the Northern Hemisphere, where solar resources typically peak from May to July and reach their minimum between November and January. In the figure, darker colors indicate higher normalized daily average power, whereas larger markers correspond to regions with stronger solar irradiance.

The normalized daily average power for each plant is defined as

(6) $$P_{\mathrm{norm}}=\frac{P_{\mathrm{day}}}{P_{\mathrm{rated}}}$$

where $P_{\mathrm{rated}}$ is the rated capacity of the plant and $P_{\mathrm{day}}$ is the yearly mean of the daily average power:

(7) $$P_{\mathrm{day}}=\frac{1}{N_{\mathrm{day}}}\sum_{d=1}^{N_{\mathrm{day}}}\left(\frac{1}{N_{d}}\sum_{i=1}^{N_{d}}P_{di}\right)$$

where $P_{di}$ denotes the power output at the *i*-th time step of day *d*, $N_{d}$ is the number of time steps within day *d*, and $N_{\mathrm{day}}$ is the total number of days in the evaluation period.

Regions with higher annual solar irradiance generally exhibit larger normalized daily average power values, indicating superior PV performance. However, this relationship is not strictly proportional across all sites. For instance, Station 04, located in an area with abundant solar radiation, shows the highest normalized daily average power, whereas Station 09 yields a comparatively lower output despite similar irradiance levels. Such discrepancies imply that PV generation performance depends not only on irradiance but also on other environmental and operational factors such as cloud cover, ambient temperature, humidity, and elevation.

**Figure 2:**
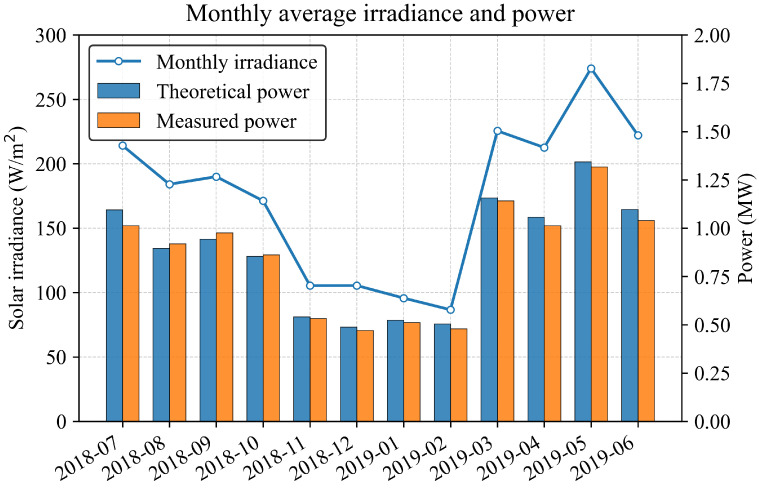
Monthly variations of average solar irradiance, theoretical power, and measured power at Station00.

**(2) Long-term (seasonal) characteristics:** The PV output exhibits distinct seasonal variability primarily governed by solar geometry and climatic conditions. To illustrate this relationship, Figure 2 presents the monthly variations of average solar irradiance, theoretical power, and measured power at Station00. The upper panel shows the monthly mean solar irradiance (blue line), while the lower panel compares the corresponding monthly average theoretical and measured power outputs (blue and orange bars, respectively). It can be observed that irradiance levels remain low during the winter months (December–February), leading to reduced theoretical and actual power generation. As the solar altitude increases in spring, irradiance gradually rises, reaching its maximum during May and June, which correspond to the highest power outputs in both the theoretical and measured series. This pattern confirms the strong seasonal dependence of PV generation on solar irradiance: higher irradiance yields greater power generation potential, whereas lower irradiance limits the available energy output. Additionally, the measured power consistently remains slightly below the theoretical estimate, reflecting real-world losses caused by module temperature effects, dust accumulation, and inverter inefficiencies.

**Figure 3:**
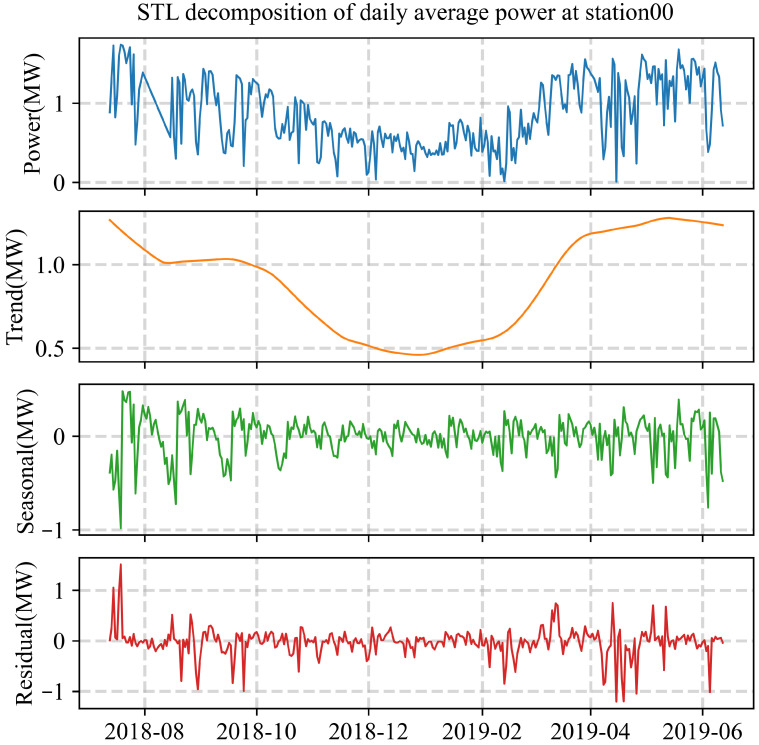
STL decomposition of daily average power at Station00. The four panels show the original series, trend, seasonal, and residual components, respectively.

To further quantify the long-term trend and periodicity of PV generation, the measured daily average power of Station00 is decomposed into three components—trend, seasonal, and residual—using the Seasonal-Trend-Loess (STL) method, as shown in Figure 3. The decomposition model can be expressed as

(8) $$P_{\mathrm{meas}}\left(t\right)=T\left(t\right)+S\left(t\right)+R\left(t\right)$$

where T(t), S(t), and R(t) represent the trend, seasonal, and residual components, respectively. The seasonal component S(t) mainly captures the periodic effects induced by solar inclination and daylight duration, whereas T(t) and R(t) describe the slow variation and random fluctuations of the PV output.

The decomposition results clearly reveal the dominant temporal patterns of PV output across multiple time scales. All PV and meteorological series are recorded at a 15-min resolution; accordingly, “day-ahead” forecasting in this study refers to predicting the full next-day PV profile with 96 time steps. The first panel in Figure 3 displays the original daily average power series, which shows pronounced seasonal variation with higher outputs in summer and lower ones in winter, consistent with the solar irradiance pattern. The second panel presents the long-term trend component T(t) (orange line), which reflects the slow evolution of PV generation after removing periodic and random fluctuations. A slight upward trend is observed, mainly associated with intra-annual variations in solar geometry and Earth–Sun distance, rather than changes in system operation or maintenance. The third panel depicts the seasonal component S(t) (green line), representing the recurrent periodic pattern of PV generation that corresponds closely to the annual solar geometry and daylight cycle. The bottom panel illustrates the residual component R(t) (red line), which contains high-frequency, small-amplitude variations arising from transient meteorological disturbances such as cloud passage, irradiance fluctuations, humidity changes, and temperature variations.

Overall, the STL analysis demonstrates that the PV output at Station00 exhibits a strong annual periodicity, with the trend and seasonal components closely following the irradiance variation. The residual fluctuations highlight the stochastic nature of PV generation, which is largely governed by fast-changing meteorological variables.

**Figure 4:**
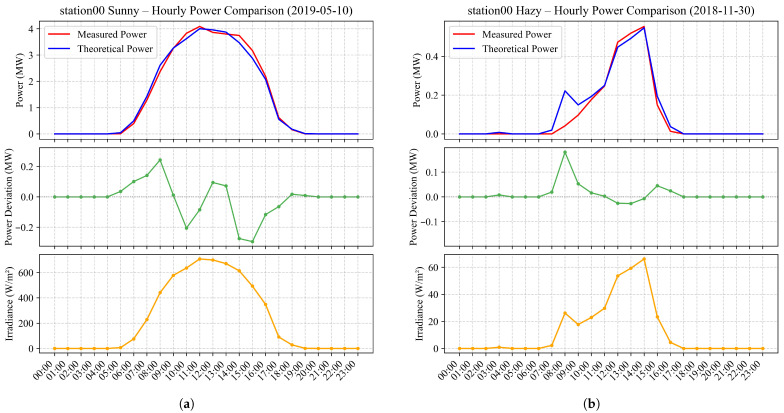
Typical PV power curves under different weather conditions. (**a**) Typical PV power curve on a sunny day. (**b**) Typical PV power curve on a hazy day.

**(3) Short-term (diurnal) characteristics:** On the diurnal scale, PV output fluctuates according to solar altitude, atmospheric turbidity, and transient cloud movements. In Figure 4, under clear-sky conditions, the power curve follows a smooth bell-shaped profile, rising rapidly after sunrise, peaking near noon, and then symmetrically decreasing towards sunset. Conversely, under cloudy or hazy conditions, the power curve exhibits multiple peaks and irregular oscillations due to intermittent irradiance variations.

Overall, this three-part analysis—covering geographical distribution and long-term seasonal and short-term diurnal characteristics—provides a comprehensive understanding of PV generation dynamics. The identified temporal patterns and environmental dependencies form the physical foundation for the data-driven forecasting framework discussed in Section 3.

## 3. Structure of the Attention-Enhanced Multivariate CNN–LSTM Model with Spatial Downscaling

### 3.1. Attention-Enhanced CNN–LSTM Architecture

**Figure 5:**
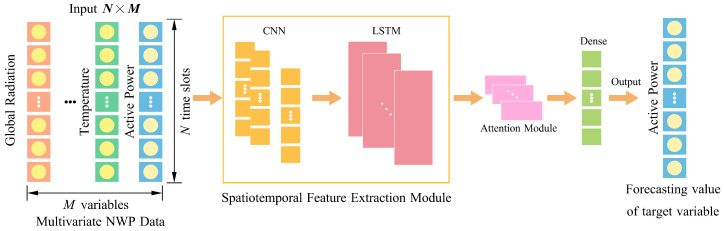
Overview of the proposed forecasting model architecture.

The overall structure of the proposed prediction model, which integrates convolutional neural networks (CNN), long short-term memory (LSTM) networks, and an attention mechanism, is illustrated in Figure 5. The architecture is designed to jointly capture local temporal patterns, long-range temporal dependencies, and dynamic feature importance. It consists of three major components: a short-term temporal module based on CNN, a long-term temporal module implemented through stacked LSTM layers, and a channel-wise attention module that adaptively recalibrates multivariate input features.

To provide a clearer understanding of the model, each module is described in detail below, highlighting its specific role in extracting spatio-temporal representations from photovoltaic (PV) and meteorological data.

(1) Convolutional neural networks (CNN)

**Figure 6:**
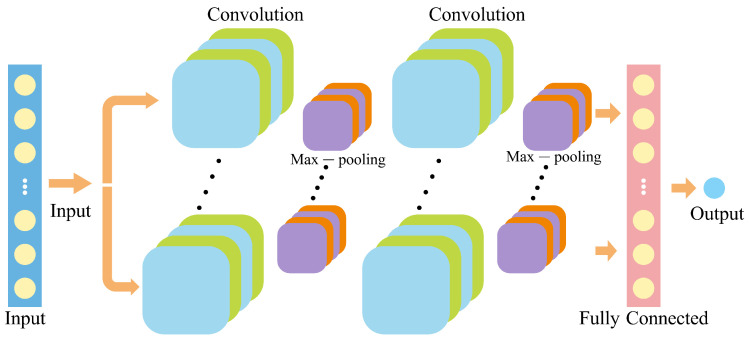
Architecture of the CNN module.

The CNN module acts as the first-stage feature extractor, responsible for capturing short-term temporal dependencies and localized fluctuations in the multivariate PV–NWP input sequence [46]. As illustrated in Figure 6, the one-dimensional convolution operation applies multiple learnable filters to the input matrix

$X\in R^{M\times Z}$, where *M* denotes the number of variables and *Z* represents the number of time steps:

(9) $$H_{\mathrm{CNN}}\left(c,t\right)=\sigma \;\left(\sum_{m=1}^{M}\sum_{k=0}^{K-1}W_{c,m,k}\;X\left(m,t-k\right)+b_{c}\right)$$

where $W_{c,m,k}$ and $b_{c}$ denote the convolution kernel and bias of the *c*-th filter, *K* represents the kernel size, and σ(·) is a nonlinear activation function (ReLU in this work). The resulting CNN feature maps $H_{\mathrm{CNN}}\in R^{C\times (Z-K+1)}$ contain rich high-frequency patterns associated with irradiance fluctuations, temperature perturbations, and short-term power ramps, where *C* denotes the number of convolutional filters.

(2) Long short-term memory networks (LSTM)

**Figure 7:**
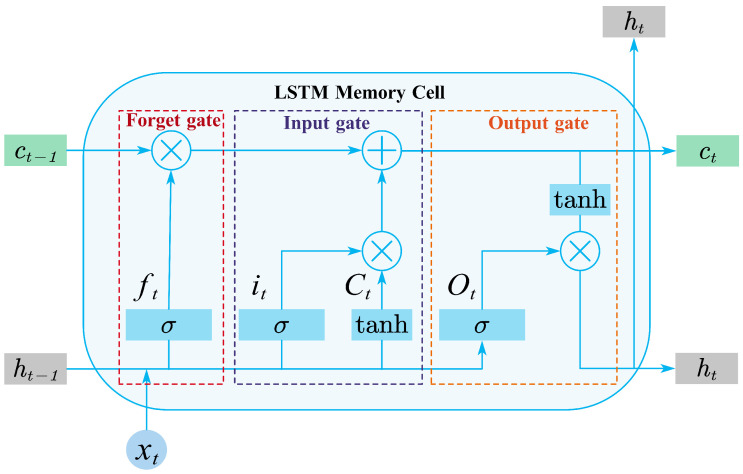
Structure of the long short-term memory (LSTM) network.

The CNN features are subsequently fed into a stacked LSTM network to model long-term temporal dependencies across the entire input horizon, as illustrated in Figure 7. The LSTM cell regulates the information flow through three gating mechanisms—forget, input, and output gates—which enable the model to capture slow-varying transitions caused by solar geometry, cloud movement, and daily irradiance cycles [47].

Given the input $x_{t}$, the previous hidden state $h_{t-1}$, and the previous cell state $c_{t-1}$, the LSTM updates are computed as follows.

**Forget gate:**

(10) $$f_{t}=\sigma \left(W_{f}\cdot \left[h_{t-1},x_{t}\right]+b_{f}\right)$$

**Input gate and candidate cell state:**

(11) $$\begin{array}{cc} i_{t} & =\sigma \left(W_{i}\cdot \left[h_{t-1},x_{t}\right]+b_{i}\right) \end{array}$$

(12) $$\begin{array}{cc} {\tilde{c}}_{t} & =\tanh\left(W_{c}\cdot \left[h_{t-1},x_{t}\right]+b_{c}\right) \end{array}$$

**Cell state update:**

(13) $$c_{t}=f_{t}⊙c_{t-1}+i_{t}⊙{\tilde{c}}_{t}$$

**Output gate and hidden state:**

(14) $$\begin{array}{cc} o_{t} & =\sigma \left(W_{o}\cdot \left[h_{t-1},x_{t}\right]+b_{o}\right) \end{array}$$

(15) $$\begin{array}{cc} h_{t} & =o_{t}⊙\tanh\left(c_{t}\right) \end{array}$$

Here, $f_{t}$, $i_{t}$, and $o_{t}$ denote the forget, input, and output gates, respectively; ${\tilde{c}}_{t}$ is the candidate cell state; and $h_{t}$ is the updated hidden state. The final hidden vector $h_{T}$ serves as a global temporal representation used by the subsequent attention and prediction layers.

(3) Attention mechanism

**Figure 8:**
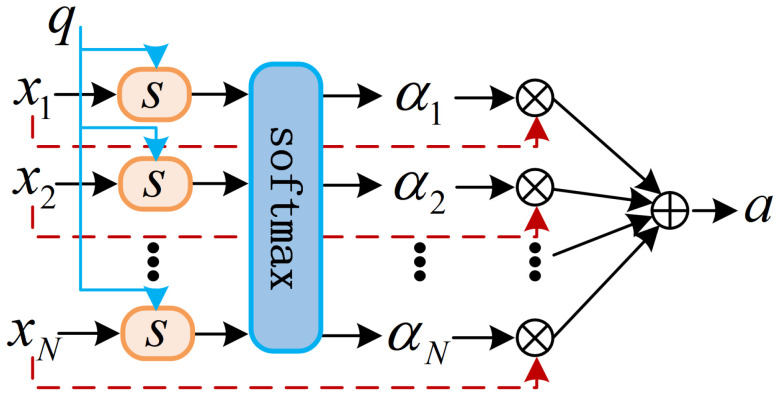
Structure of the attention module.

To further enhance the discriminative capability of temporal features, an attention mechanism is applied after the LSTM module (Figure 8). This mechanism computes a relevance score for each hidden state and uses softmax normalization to generate attention weights that highlight the most informative time steps [48]. Similar attention-enhanced recurrent architectures have been reported to improve temporal representation in energy-related time-series learning, such as battery health prediction for EV fleets  [49].

Given the LSTM output sequence $h_{1},h_{2},\ldots ,h_{T}$ and a query vector q, the attention score for time step *t* is computed as

(16) $$s_{t}=\tanh\;\left(W_{s}\cdot \left[h_{t},q\right]+b_{s}\right)$$

where [·] denotes vector concatenation. The query vector q is implemented as a learnable context vector that is randomly initialized and jointly optimized with the attention parameters during training. It serves as a global temporal query to adaptively weight the LSTM hidden states according to their relevance for day-ahead PV power forecasting.

The attention weight $\alpha_{t}$ is then obtained through a softmax function:

(17) $$\alpha_{t}=\frac{\exp(s_{t})}{\sum_{j=1}^{T}\exp\left(s_{j}\right)}$$

The final context vector is computed as a weighted sum of the hidden states:

(18) $$a=\sum_{t=1}^{T}\alpha_{t}h_{t}$$

The resulting context vector a emphasizes the most relevant temporal components while suppressing noisy or redundant information, thereby improving the robustness and accuracy of the day-ahead PV power forecasts.

### 3.2. Multivariate Input Fusion

Different input–output configurations have been applied in photovoltaic (PV) power forecasting, ranging from single-input single-output structures to multi-input multi-output models [50]. However, PV power generation is strongly influenced by multiple external environmental factors, including solar irradiance, temperature, humidity, wind speed, wind direction, and atmospheric pressure. Relying solely on historical PV power limits the model’s ability to describe nonlinear variations induced by weather conditions.

**Figure 9:**
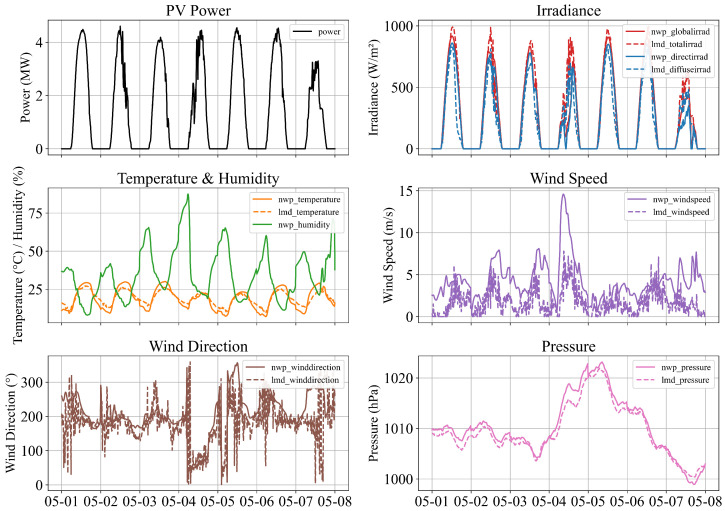
Multivariate PV and meteorological features within the selected period.

Figure 9 shows the temporal evolution of PV power and five groups of meteorological variables, using the one-week period from 1–8 May 2019 as an illustrative example: irradiance components, temperature and humidity, wind speed, wind direction, and atmospheric pressure. Solid lines represent NWP forecasts, whereas dashed lines indicate on-site measurements. Irradiance—especially the global and direct components—exhibits the strongest temporal consistency with PV output. Temperature, humidity, and wind variables reflect slower environmental variations, whereas pressure captures broader atmospheric trends. These grouped features highlight the multi-scale dependencies influencing PV generation and motivate the integration of multivariate inputs into the forecasting model.

To fully capture the multi-factor influence on PV generation, this study adopts a multivariate input fusion strategy. All relevant environmental predictors extracted from numerical weather prediction (NWP) data, together with historical PV power measurements, are combined to form a unified multivariate input vector. The input at each time step is defined as

(19) $$x_{t}=\left[\;{\mathrm{NWP}}_{t},\;P_{t}\;\right]\in R^{M}$$

where ${\mathrm{NWP}}_{t}$ includes forecasted radiation, temperature, humidity, wind, and pressure, and $P_{t}$ denotes the actual PV power.

The multivariate input sequence is given by

(20) $$X=\left[\;x_{1},x_{2},\ldots ,x_{T}\;\right]\in R^{M\times T}$$

with *M* being the number of input variables. This fusion of meteorological and power features enriches the model’s representational capacity and enables it to learn both direct and indirect environmental impacts on PV output.

Table 1 summarizes the detailed composition of the fused input set used in this study.

**Figure 10:**
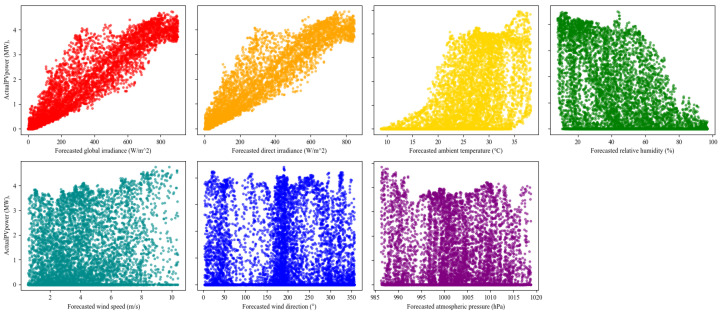
Scatter-Based Relationship Between Forecasted Meteorological Variables and Measured PV Power.

To provide interpretability for the meteorological inputs, Figure 10 shows scatter plots between the measured PV power P(t) and each forecasted meteorological predictor $x_{k}\left(t\right)$. Irradiance-related variables exhibit the strongest positive dependence on PV power, while humidity tends to be negatively associated and wind/pressure variables show weaker and more dispersed relationships. We further quantify feature relevance on daytime samples to avoid nighttime bias. Let $D_{\mathrm{day}}=\left\{t|\mathrm{GHI}(t)>\tau \right\}$. The Pearson correlation between P(t) and $x_{k}\left(t\right)$ is

(21) $$r_{k}=\frac{\sum_{t\in D_{\mathrm{day}}}\left(x_{k}\left(t\right)-{\bar{x}}_{k}\right)\left(P\left(t\right)-\bar{P}\right)}{\sqrt{\sum_{t\in D_{\mathrm{day}}}{\left(x_{k}\left(t\right)-{\bar{x}}_{k}\right)}^{2}}\sqrt{\sum_{t\in D_{\mathrm{day}}}{\left(P\left(t\right)-\bar{P}\right)}^{2}}}$$

and the normalized relevance weight is defined as

(22) $$\begin{array}{cc} w_{k} & =\frac{|r_{k}|}{\sum_{j=1}^{K}\left|r_{j}\right|},\;\sum_{k=1}^{K}w_{k}=1 \end{array}$$

**Figure 11:**
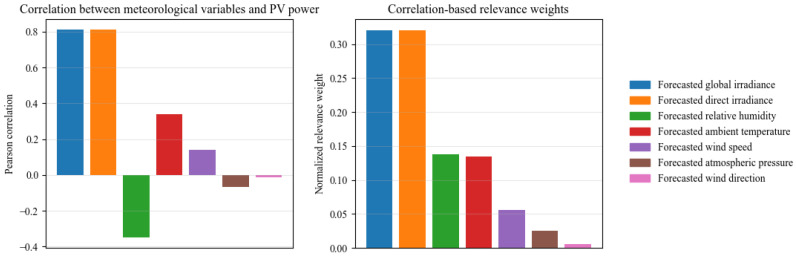
Pearson Correlation and Correlation-Based Relevance Weights of Meteorological Predictors for PV Power.

Figure 11 reports $r_{k}$ and $w_{k}$, confirming that irradiance predictors dominate PV power variations, whereas humidity/temperature are secondary and wind/pressure variables are less informative. This analysis supports the use of attention, which helps the model emphasize informative drivers and suppress weakly relevant inputs during representation learning.

### 3.3. Spatial Downscaling Module

**Figure 12:**
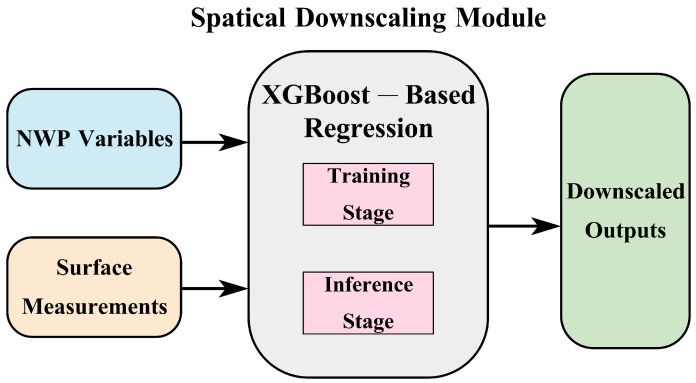
Overall workflow of the spatial downscaling module.

Although numerical weather prediction (NWP) data are used as the primary meteorological inputs to the forecasting model described in Section 3.2, their coarse spatial resolution (typically on the order of several kilometers) cannot fully represent the fine-scale atmospheric variability experienced by a megawatt-scale PV plant. As a result, raw NWP variables may deviate from the true local meteorological state, introducing systematic biases and degrading forecasting reliability. To mitigate this limitation, we introduce a spatial downscaling module that refines large-scale NWP fields into high-fidelity, site-specific meteorological inputs, as illustrated in Figure 12.

Unlike the forecasting model, which directly uses NWP-based predictors as meteorological inputs, the downscaling module learns a cross-site mapping by leveraging multi-site ground measurements together with the corresponding NWP variables, thereby correcting spatial biases in a data-driven yet operationally feasible manner [51]. Once trained, the model can infer refined, high-resolution meteorological variables for the target station using NWP inputs alone, enabling localized meteorological reconstruction even when local measurements are unavailable [52].

Let the *i*-th PV station be located at $\left({\mathrm{lon}}_{i},{\mathrm{lat}}_{i}\right)$, and define the multivariate NWP-driven input vector at time *t* as

(23) $$X_{i,t}=\left[{\mathrm{lon}}_{i},\;{\mathrm{lat}}_{i},\;{\mathrm{hour}}_{t},\;x_{i,t}^{\left(1\right)},\;x_{i,t}^{\left(2\right)},\;\ldots ,\;x_{i,t}^{\left(M\right)}\right]$$

where ${\mathrm{hour}}_{t}$ encodes the diurnal cycle and $x_{i,t}^{\left(m\right)}$ denotes the *m*-th NWP variable (temperature, humidity, wind speed, wind direction, pressure, global irradiance, and direct irradiance).

For stations with available surface measurements (station01–station09), the corresponding observed meteorological vector is defined as

(24) $$Y_{i,t}=\left[y_{i,t}^{\left(1\right)},\;y_{i,t}^{\left(2\right)},\;\ldots ,\;y_{i,t}^{\left(N\right)}\right]$$

where $y_{i,t}^{\left(n\right)}$, n=1,…,N, denotes the *n*-th observed variable (temperature, wind speed, wind direction, pressure, global irradiance, and direct irradiance).

The proposed integration strategy is fundamentally characterized by a leakage-free, cross-station transfer coupling between spatial downscaling and day-ahead PV forecasting. Specifically, the downscaling model is trained using measurement-based pairs from surrounding stations only and is then transferred to the target station using NWP inputs alone; the target station contributes no measurement pairs during downscaling training. The resulting downscaled site-level meteorological variables serve as the exclusive exogenous drivers for the attention-enhanced CNN–LSTM forecaster at the target station, such that improvements in meteorological reconstruction can be directly propagated to forecasting accuracy under a unified and leakage-free protocol [52].

While neural and physics-guided downscaling approaches are common, we cast downscaling as a tabular, multi-output regression from location/time encodings and heterogeneous NWP predictors to site-level meteorological variables. In this setting, XGBoost offers strong nonlinear modeling with minimal preprocessing and robust performance on mixed predictors, typically requiring fewer design and tuning choices than neural downscalers [53]. Since physically informed post-processing/downscaling methods often rely on additional high-resolution geographic/physical descriptors and/or dedicated physical parameterizations that are not consistently available across PVOD sites, XGBoost is adopted as a pragmatic and reproducible choice under our dataset setting [51].

The spatial downscaling procedure consists of two stages:

**(1) Training stage:** Stations with available measurements (station01–station09) provide paired samples $\left(X_{i,t},Y_{i,t}\right)$, enabling the model to learn the mapping from coarse-resolution NWP features to localized observations.

**(2) Inference stage:** For the target station (e.g., station00), which provides no measurements during training, the trained model takes only its location, time-of-day, and raw NWP features as input and outputs refined high-resolution meteorological estimates:

(25) $${\hat{Y}}_{t}=f_{\theta }\left(X_{t}\right)$$

where $f_{\theta }$ denotes the learned XGBoost-based multi-output regression model.

These refined meteorological inputs provide a high-resolution representation of the local atmospheric state and are subsequently integrated into the CNN–LSTM–Attention forecasting model to enhance the spatial consistency and accuracy of the day-ahead PV power forecasts.

### 3.4. Day-Ahead Forecasting Modeling Framework

**Figure 13:**
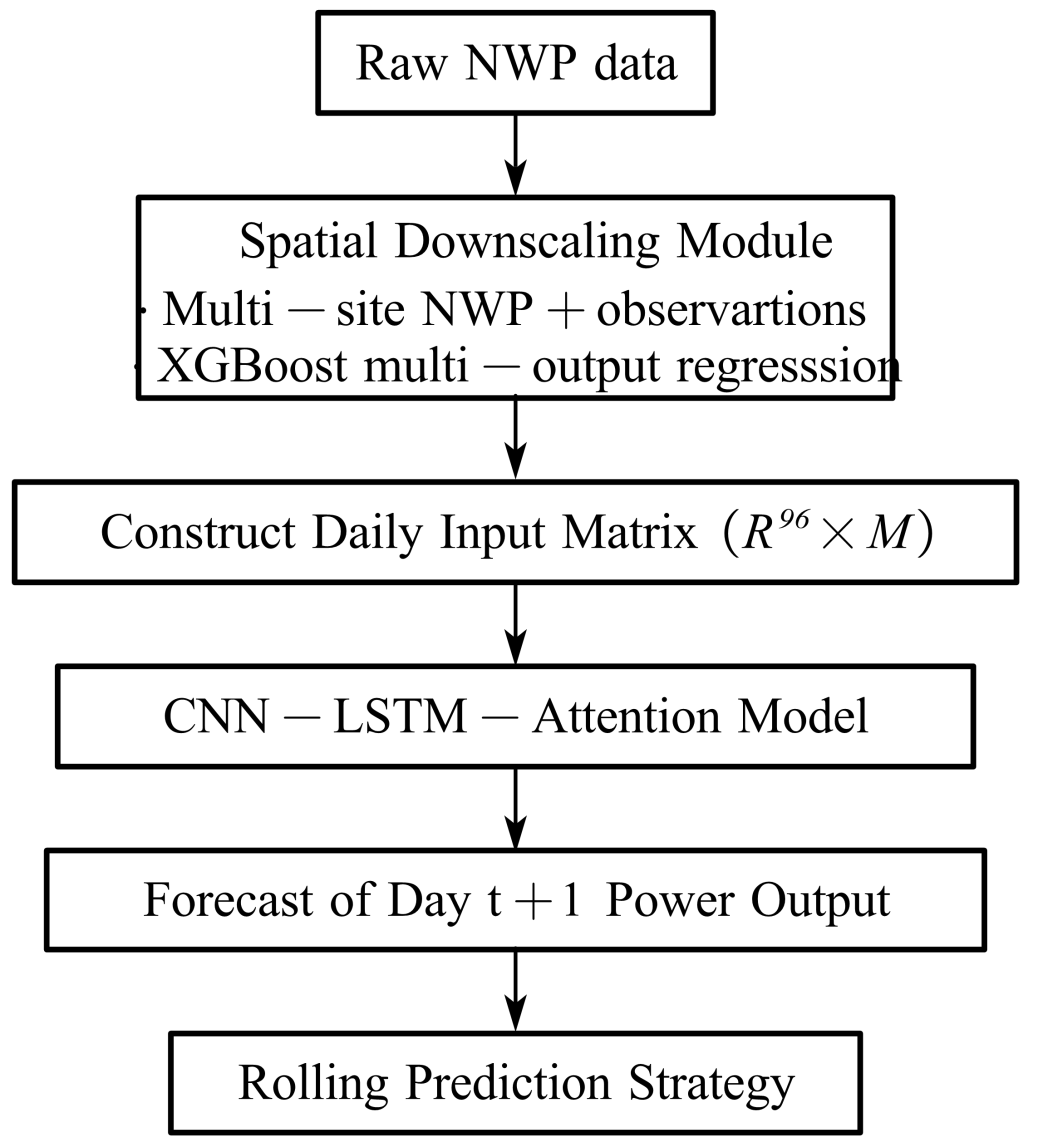
Overall rolling day-ahead PV forecasting framework.

The proposed framework performs rolling day-ahead PV power forecasting based on high-resolution meteorological inputs obtained from the spatial downscaling module in Section 3.3. Specifically, the coarse-resolution NWP fields for day *t* are first refined into site-specific meteorological variables through the XGBoost-based downscaling model. These downscaled features, together with the historical PV power measurements for day *t*, are then organized into a multivariate input matrix and fed into the CNN–LSTM–Attention forecasting network to generate the complete 96-point PV power profile for day t+1. The overall workflow of the proposed forecasting framework is illustrated in Figure 13.

Let each day consist of 96 time steps with a 15-min resolution. After spatial downscaling, the multivariate feature matrix of day *t* is defined as

(26) $$X_{t}\in R^{96\times M}$$

where *M* denotes the number of input variables, which are strictly defined as the seven NWP predictors listed in Table 1 (including humidity) together with the corresponding PV power of day *t*. The day-ahead target is the PV power sequence of the following day:

(27) $$Y_{t+1}\in R^{96}$$

This leads to a one-day-input/one-day-output learning structure, in which the forecasting model is written as

(28) $${\hat{Y}}_{t+1}=f_{\theta }\left(X_{t}\right)$$

where $f_{\theta }\left(\cdot \right)$ denotes the attention-enhanced CNN–LSTM network.

During testing, the last seven days of each selected month (February, May, August, and November) are used as rolling evaluation windows. For each day in this period, the framework first applies the spatial downscaling module to the raw NWP inputs of day *t*, constructs $X_{t}$, and then generates the day-ahead forecast:

(29) $$X_{t}\to {\hat{Y}}_{t+1},\;X_{t+1}\to {\hat{Y}}_{t+2},\;\ldots$$

This rolling strategy is consistent with practical operation, where only the current day’s information and NWP forecasts are available for next-day scheduling.

## 4. Case Study

### 4.1. Experimental Data

This study uses the publicly available PVOD v1.0 photovoltaic power dataset [54], hosted on the Science Data Bank. PVOD provides multi-source meteorological and operational measurements that are well suited to PV power forecasting, solar irradiance modelling, and grid-integration research. The dataset contains 271,968 samples at a uniform 15-min temporal resolution and is constructed from two complementary data sources: (1) numerical weather prediction (NWP) data provided by a national meteorological service, and (2) local measurements data (LMD) recorded at PV power stations. The complete list of input variables considered in this study is summarized in Table 1.

The coexistence of coarse-resolution NWP forecasts and fine-grained local measurements enables a rigorous evaluation of both the proposed spatial downscaling module (Section 3.3) and the day-ahead forecasting framework (Section 3.4). In this work, station00 is designated as the target station for forecasting, and the remaining stations (station01–station09) are used to train the downscaling model.

All features are synchronized to a 15-min interval. Four representative months—February, May, August, and November—are selected to capture diverse seasonal patterns, including winter low-irradiance periods, transitional spring conditions, summer high-irradiance periods with strong variability, and relatively stable autumn operating conditions. For each month, the last seven days serve as the test set for rolling day-ahead forecasting, whereas all preceding days are used for model training.

### 4.2. Performance Indices

To evaluate the prediction accuracy and trend consistency of the proposed PV power forecasting framework, four performance indices are employed: root mean square error (RMSE), mean absolute error (MAE), Pearson correlation coefficient (*r*), and accuracy (CR). These metrics jointly quantify the magnitude of prediction errors, the average deviation level, the consistency of temporal patterns, and the overall forecasting skill.

(1) Root mean square error (RMSE)

(30) $$\mathrm{RMSE}=\sqrt{\frac{1}{N}\sum_{i=1}^{N}{\left(\frac{P_{i}^{\mathrm{pred}}-P_{i}^{\mathrm{true}}}{C_{\mathrm{rated}}}\right)}^{2}}$$

(2) Mean absolute error (MAE)

(31) $$\mathrm{MAE}=\frac{1}{N}\sum_{i=1}^{N}\left|\frac{P_{i}^{\mathrm{pred}}-P_{i}^{\mathrm{true}}}{C_{\mathrm{rated}}}\right|$$

In both RMSE and MAE, *N* denotes the total number of samples and $C_{\mathrm{rated}}$ is the rated capacity of the PV plant, so that the errors are expressed in per-unit form.

(3) Pearson correlation coefficient (*r*)

(32) $$r=\frac{\sum_{i=1}^{N}\left(P_{i}^{\mathrm{true}}-\bar{P^{\mathrm{true}}}\right)\left(P_{i}^{\mathrm{pred}}-\bar{P^{\mathrm{pred}}}\right)}{\sqrt{\sum_{i=1}^{N}{\left(P_{i}^{\mathrm{true}}-\bar{P^{\mathrm{true}}}\right)}^{2}\sum_{i=1}^{N}{\left(P_{i}^{\mathrm{pred}}-\bar{P^{\mathrm{pred}}}\right)}^{2}}}$$

where $\bar{P^{\mathrm{true}}}$ and $\bar{P^{\mathrm{pred}}}$ denote the sample means of the true and predicted PV power, respectively. The coefficient *r* measures the linear consistency between the predicted and actual time series.

(4) Accuracy (CR)

(33) CR=(1−RMSE)×100%

Here, CR is not intended as a conventional accuracy metric. Instead, it is used as an error-to-score indicator for intuitive interpretation. Specifically, RMSE is computed on the normalized PV power series. Therefore, CR can be interpreted as the percentage score relative to the zero-error case, where a larger CR indicates a smaller forecasting error. In this work, CR is reported only as a supplementary index, while RMSE and MAE remain the primary error measures.

Together, these four indices provide a balanced assessment of both numerical accuracy and temporal correlation between the predicted and actual PV power profiles.

### 4.3. Experimental Design

To systematically evaluate the effectiveness of the proposed forecasting framework, three groups of experiments are conducted. These experiments follow a progressive evaluation strategy: (1) validating the spatial downscaling module, (2) comparing different forecasting input modes, and (3) comparing different model architectures. All forecasting tasks follow the rolling day-ahead procedure introduced in Section 3.4 and use identical training configurations to ensure a fair comparison.

(1) Comparison of NWP errors before and after spatial downscaling

The first experiment evaluates the effectiveness of the spatial downscaling module (Section 3.3). For the target station (station00), we compare the raw NWP variables with the downscaled estimates for five locally measurable variables (temperature, wind speed, pressure, global irradiance, and diffuse irradiance). To better justify the choice of XGBoost, we additionally include two representative downscaling baselines under the same protocol: (i) a neural baseline implemented as a multi-output multilayer perceptron (MLP) with shared hidden layers and a five-dimensional output head, trained on the same inputs and targets; and (ii) a physically inspired statistical correction baseline that applies per-variable bias correction learned from the training stations and transferred to the target station. All methods strictly follow the leakage-free setting—no measurement-based pairs from the target station are used during downscaling training—and hyper-parameters are selected using validation folds constructed from the surrounding stations. The improvement in accuracy is quantified using the performance indices introduced in Section 4.2, namely RMSE, MAE, the Pearson correlation coefficient, and accuracy (CR). This experiment assesses whether the downscaling model can effectively reduce spatial biases and provide higher-fidelity meteorological drivers for the subsequent day-ahead PV forecasting task.

(2) Comparison of forecasting modes with different input features

The second experiment evaluates three forecasting modes that differ in their input feature composition:

- Historical power only: A univariate baseline model that uses only past PV power measurements.
- Historical power + original NWP: A multivariate forecasting model that incorporates raw NWP variables.
- Historical power + downscaled NWP: A forecasting model that uses high-resolution meteorological inputs generated by the spatial downscaling module.

This experiment quantifies the incremental benefits introduced by (1) adding large-scale atmospheric forecasts and (2) refining these forecasts through spatial downscaling. All three modes are evaluated under an identical CNN–LSTM–Attention forecasting architecture to ensure that performance differences arise solely from the quality of the input information.

(3) Comparison of forecasting model architectures

The third experiment isolates the effect of model structure. Using the same enhanced input configuration (historical power + downscaled NWP), three neural network architectures are compared:

- LSTM;
- CNN–LSTM;
- CNN–LSTM–Attention (proposed).

This experiment evaluates the respective contributions of temporal feature extraction (LSTM), local spatio-temporal encoding (CNN), and channel-wise relevance reweighting (attention). The comparison highlights how architectural enhancements improve the forecasting capability when the models are supplied with the highest-quality meteorological inputs.

(4) Unified evaluation protocol

All forecasting models are evaluated under a unified protocol using the performance metrics defined in Section 4.2, namely RMSE, MAE, the Pearson correlation coefficient, and accuracy (CR). These metrics jointly assess pointwise accuracy and the temporal consistency of the predicted PV power series. To ensure a fair comparison, all models are trained using identical hyperparameter settings, including the Adam optimizer and a composite loss function, so that performance differences originate solely from the input configuration or model architecture.

For reproducibility, the key training hyperparameters are fixed across all forecasting models. Specifically, the input/output sequence lengths are set to 96/96 (i.e., one-day-ahead prediction with 96 time steps). The forecasting network uses a Conv1D front-end (8 to 32, kernel size 7) followed by an SE block and an LSTM backbone (hidden size 128, 3 layers, dropout 0.2). The models are optimized using Adam with a learning rate of $1\times 10^{-4}$ and weight decay $1\times 10^{-5}$, with a batch size of 32 and a maximum of 5000 training epochs. The composite loss is defined as a weighted combination of MSE and MAE with α=0.9 (L=α·MSE+(1−α)·MAE). All models share the same preprocessing pipeline, where the PV power and meteorological inputs are normalized before training and the outputs are inverse-transformed for evaluation.

In addition, the spatial downscaling module is trained with a consistent XGBoost configuration. The downscaler is implemented as a MultiOutputRegressor of XGBRegressor, i.e., one independent XGBoost model per output variable, with n_estimators = 100 and random_state = 42 (all other hyper-parameters kept at default settings). The input features include station location (latitude/longitude), hour-of-day, and NWP predictors. A strict leakage-free cross-station protocol is used: only station01–station09 provide supervised pairs, and the trained model is transferred to station00 using NWP inputs only to generate station-specific meteorological features for forecasting.

All experiments are conducted on a workstation equipped with an Intel Core i7-12700KF processor (3.60 GHz), 32 GB RAM, and an NVIDIA RTX 3060 GPU with 12 GB of dedicated memory, running a 64-bit Windows operating system. The forecasting models are implemented in Python 3.10 using the PyTorch 2.1 deep learning framework, developed in PyCharm 2024, while data preprocessing and visualization are performed with NumPy 1.23.5, Pandas 1.5.3, and Matplotlib 3.7.0. This consistent computing environment ensures reproducible training behaviour and reliable performance comparisons across all model variants.

## 5. Result Analysis

This section presents a systematic evaluation of the experimental results, covering three aspects: the effectiveness of the spatial downscaling module, the comparison of forecasting modes under different input configurations, and the comparison of different forecasting model architectures. All results are assessed using the four performance indices defined in Section 4.2, namely RMSE, MAE, the Pearson correlation coefficient, and accuracy.

### 5.1. Effectiveness of Spatial Downscaling

**Figure 14:**
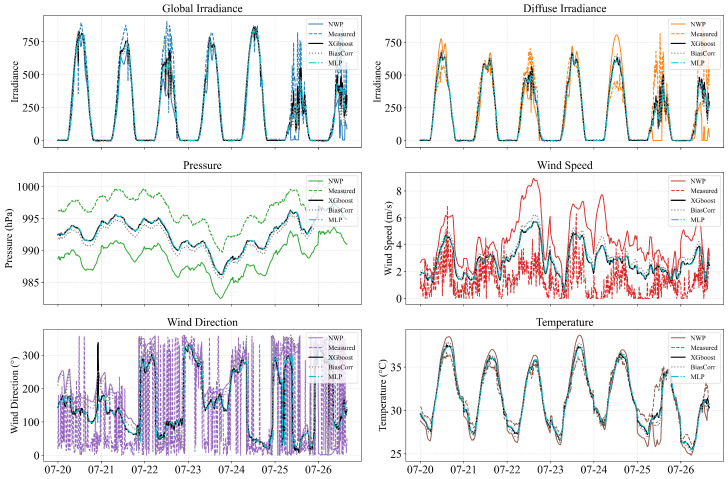
Time-series comparison of NWP, measured, and downscaled meteorological variables.

To evaluate the effectiveness of the proposed spatial downscaling module, the raw NWP data for station00 are compared with their downscaled counterparts produced by three different downscaling methods over a selected multi-day period. As shown in Figure 14, the downscaled meteorological variables exhibit substantially improved alignment with the on-site measurements across all five observable quantities (temperature, wind speed, pressure, global irradiance, and diffuse irradiance). Compared with the original NWP forecasts—which often deviate substantially from local dynamics—the downscaled series better capture short-term fluctuations in the observed variables and display smoother, more realistic temporal behaviour for humidity and wind direction.

**Figure 15:**
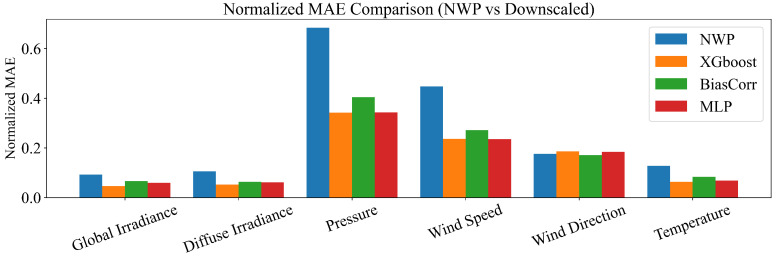
Normalized MAE comparison between raw NWP and downscaled results.

**Figure 16:**
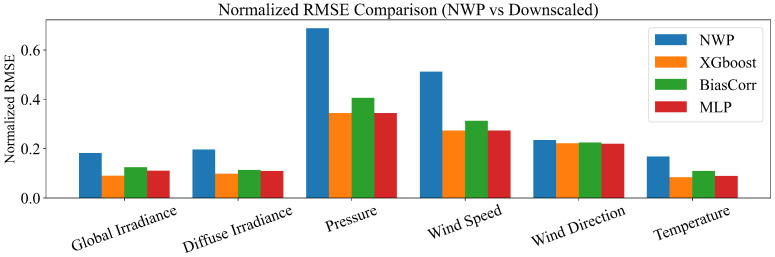
Normalized RMSE comparison between raw NWP and downscaled results.

Quantitative improvements are summarized in Figure 15 and Figure 16. Overall, all three downscaling approaches (XGBoost, BiasCorr, and MLP) reduce both NMAE and NRMSE relative to the raw NWP fields, with XGBoost and MLP generally achieving the lowest errors, followed by BiasCorr. The most pronounced gains are observed for irradiance-related variables (global and diffuse irradiance), where the normalized errors are reduced by roughly 40%–55% compared with raw NWP. Substantial improvements are also obtained for pressure and wind speed, indicating that the downscaling procedure effectively corrects spatial bias not only in radiative forcing but also in dynamic meteorological fields. In contrast, wind direction exhibits comparatively limited error reduction across methods, which is consistent with the intrinsic circular nature and higher uncertainty of directional measurements.

Overall, these results confirm that the downscaling module can substantially enhance the spatial fidelity of meteorological inputs at the station level, providing a more accurate basis for downstream forecasting tasks such as PV power prediction. It is worth noting that although the MLP baseline achieves performance comparable to XGBoost in several variables, it typically requires more elaborate architecture choices and hyper-parameter tuning to reach a stable optimum, whereas XGBoost delivers similarly strong accuracy with a simpler and more reproducible configuration in this tabular multi-output setting.

### 5.2. Comparison of Forecasting Modes with Different Input Features

This subsection compares the forecasting performance of different input-feature configurations to examine how various sources of information contribute to day-ahead PV power prediction. Three modes are evaluated: (i) using historical power alone, (ii) incorporating raw NWP predictors, and (iii) integrating spatially downscaled meteorological variables. By contrasting these modes under the same modelling framework and training settings, the analysis highlights the extent to which refined meteorological features enhance predictive accuracy and temporal consistency, particularly under seasonally variable and rapidly changing weather conditions.

**Figure 17:**
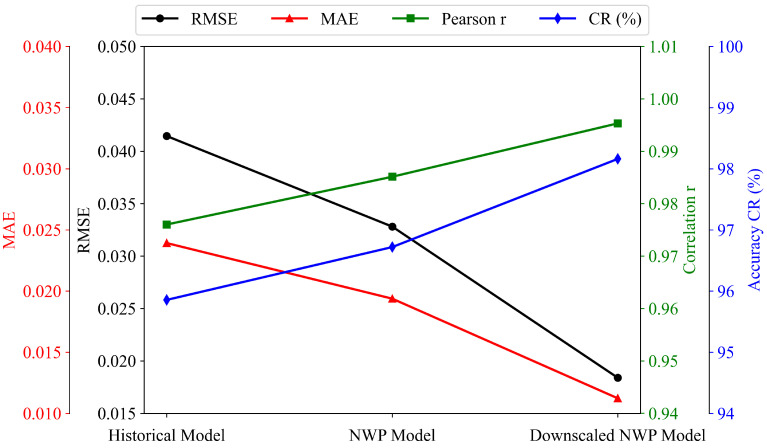
Performance comparison of three forecasting modes across four evaluation metrics.

Figure 17 shows that forecasting performance improves consistently as richer and more localized meteorological information is included. The historical-power model exhibits the largest errors, with an RMSE of approximately 0.041 and the lowest accuracy and correlation. Introducing raw NWP inputs significantly reduces both RMSE and MAE while increasing correlation, indicating that large-scale atmospheric information helps the model capture irradiance-driven fluctuations.

The best performance is obtained when downscaled NWP features are used, as shown in Table 2. RMSE and MAE drop sharply to their minimum values (approximately 0.018 and 0.012, respectively), while the correlation rises above 0.99 and the overall accuracy exceeds 98%. These results confirm that refining spatial meteorological information at the local station level is crucial for improving short-term PV forecasting.

**Figure 18:**
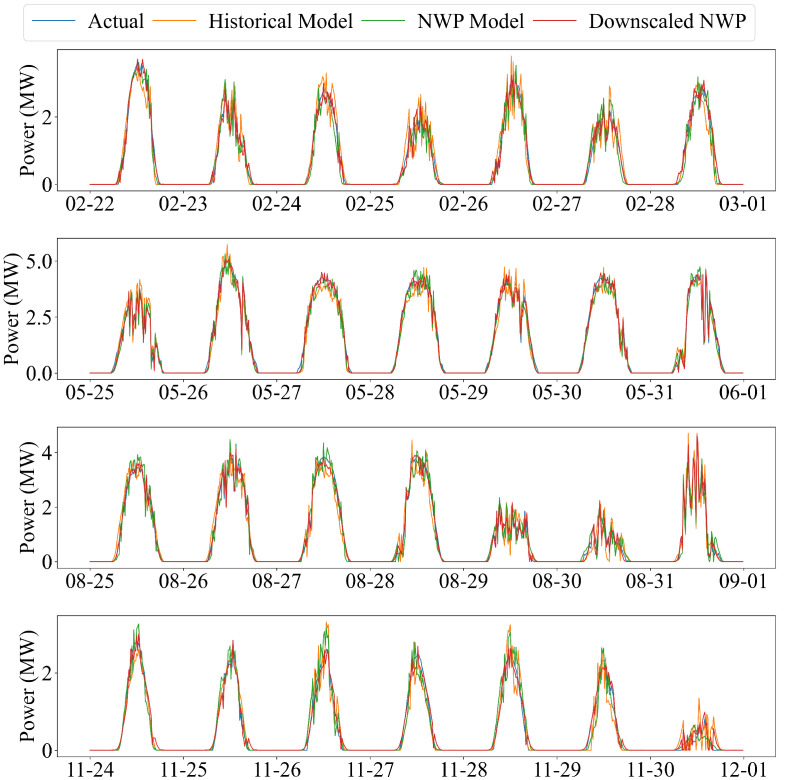
Forecasting comparison over the last seven days of four different months using the three input-feature modes.

Figure 18 further illustrates the prediction curves over the last seven days of four different months (February, May, August, and November), which serve as seasonally separated periods to assess cross-period robustness under distinct irradiance regimes. Across all four windows, the downscaled NWP model consistently produces curves that most closely follow the measured power, including both smooth clear-sky shapes and days with pronounced intra-day variability. In contrast, the raw NWP model exhibits moderate bias and timing errors, whereas the historical-power model often smooths or lags rapid ramp-up and ramp-down behaviour. The consistent ranking observed across these seasonally distinct periods qualitatively supports that spatial downscaling corrects site-specific irradiance and meteorological patterns, leading to more accurate and stable predictions over time.

Overall, the experiments demonstrate that the downscaled NWP input mode provides the most accurate, stable, and weather-adaptive forecasting performance, validating the necessity of high-resolution meteorological refinement for PV power forecasting.

### 5.3. Comparison of Forecasting Model Architectures

This subsection examines how different neural network architectures affect day-ahead PV power forecasting performance. Three models are evaluated: (i) a standard LSTM network, (ii) a hybrid convolutional–LSTM (CNN-LSTM) architecture, and (iii) an attention-enhanced CNN-LSTM (AC-LSTM). All models are trained under identical settings, and their performance is compared using four metrics—RMSE, MAE, the Pearson correlation coefficient *r*, and the accuracy (CR)—to quantify differences in error magnitude, temporal consistency, and overall predictive reliability.

**Figure 19:**
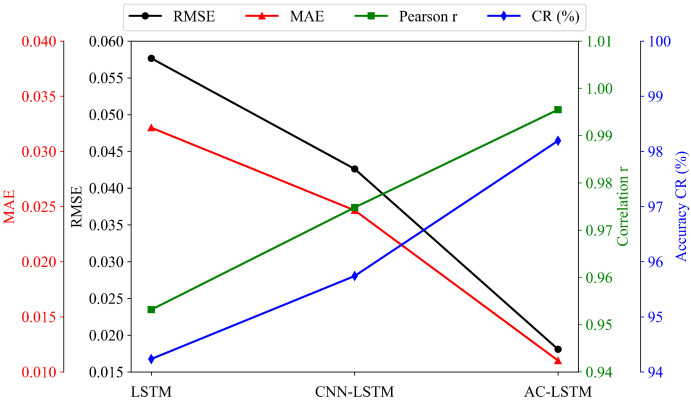
Performance comparison of three forecasting architectures across four evaluation metrics.

Figure 19 shows that forecasting performance improves consistently as more advanced architectural components are incorporated. The baseline LSTM model exhibits the largest RMSE and MAE values, reflecting its limited ability to capture complex nonlinear dependencies in irradiance-driven PV dynamics. Introducing convolutional layers in the CNN-LSTM model significantly enhances performance: RMSE and MAE drop from 0.0577 and 0.0321 to 0.0426 and 0.0247, respectively, while the correlation coefficient increases notably. This indicates that local spatio-temporal feature extraction plays an important role in enhancing temporal forecasting accuracy.

The best overall performance is obtained with the AC-LSTM model, as shown in Table 3. By integrating an attention mechanism, AC-LSTM adaptively emphasizes the most informative temporal features, resulting in the lowest RMSE (0.0181) and MAE (0.0111), together with a correlation of 0.995 and an accuracy exceeding 98%. These improvements confirm that attention-based feature reweighting substantially strengthens the model’s ability to track PV ramps, respond to abrupt cloud transitions, and generalize across diverse weather patterns.

**Figure 20:**
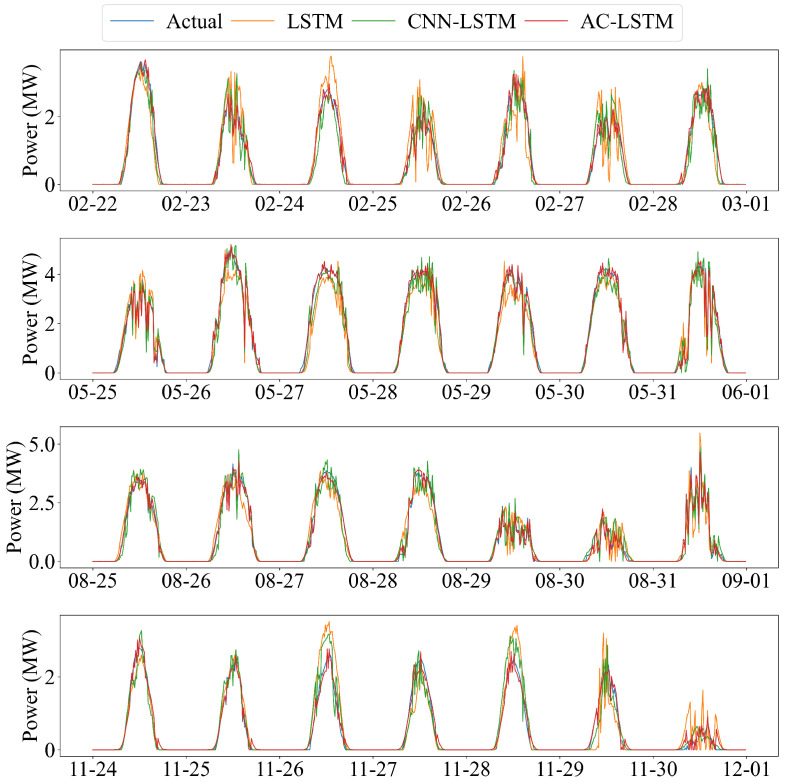
Forecasting comparison over the last seven days of four different months using the three model architectures.

Figure 20 further illustrates the forecasting sequences for four representative weeks in February, May, August, and November. Across all months and weather conditions, the AC-LSTM predictions most closely follow the measured power curves, exhibiting accurate peak alignment and minimal ramping error. The CNN-LSTM model performs moderately well but still shows distortions during highly variable periods, whereas the LSTM model often underestimates peak values and fails to replicate rapid fluctuations. These qualitative comparisons reinforce the quantitative metrics and demonstrate that attention-enhanced spatio-temporal modelling provides superior robustness and adaptability.

Overall, the experimental results demonstrate that architectural enhancement—from pure recurrence (LSTM), to spatio-temporal fusion (CNN-LSTM), and finally to attention-guided feature selection (AC-LSTM)—leads to progressively improved forecasting accuracy and stability. Among the three, the AC-LSTM model shows the strongest performance and provides the most reliable predictions under diverse meteorological conditions.

## 6. Conclusions

In this paper, a forecasting framework integrating spatially downscaled NWP data and advanced deep learning architectures has been developed for day-ahead PV power prediction. The experimental results consistently demonstrate that both refined meteorological inputs and enhanced model structures contribute significantly to forecasting accuracy.

The comparison of input modes shows that the historical-power-only model suffers from limited adaptability under changing weather conditions. Incorporating raw NWP inputs improves all error metrics, but the best performance is achieved when downscaled NWP features are used: RMSE and MAE are reduced by more than 40%, and the correlation coefficient exceeds 0.99. Time-series results across four representative weeks further confirm that downscaled NWP inputs enable the model to better track rapid morning and evening ramps that other modes fail to capture.

Regarding forecasting architectures, the CNN–LSTM and the attention-enhanced AC-LSTM both outperform the baseline LSTM in all evaluation metrics. The AC-LSTM achieves the lowest RMSE and MAE and the highest accuracy, benefiting from the complementary strengths of convolutional feature extraction, temporal modelling, and attention-based relevance weighting.

Overall, the findings confirm that spatial refinement of NWP data, combined with hybrid neural architectures, substantially enhances the robustness and precision of PV power forecasting. The proposed AC-LSTM model provides a reliable tool for operational decision-making in power system scheduling and renewable integration, and offers a solid foundation for future extensions to probabilistic forecasting and other renewable energy applications.

Several limitations should be acknowledged. First, the proposed pipeline introduces additional computational cost, particularly in the offline training stage of the downscaling module and the subsequent forecasting model; although inference is relatively lightweight once models are trained, the overall workflow requires non-negligible training time and resources. Second, the approach relies on the availability and quality of multi-station measurements to learn cross-site relationships for downscaling. When measurements are sparse, contain substantial missing values, or are affected by quality issues, the effectiveness of downscaling may be reduced. Third, transferability to regions with limited measurements or markedly different climatic and geographic conditions is not guaranteed, as domain shifts can alter both local meteorological patterns and the error characteristics of NWP predictors.

In future work, we will further investigate how the density and coverage of training stations affect the robustness of the spatial downscaling mapping, and develop practical guidelines for deploying the proposed pipeline in observation-sparse regions. In addition, extending the framework to probabilistic forecasting and exploring cross-region transfer/adaptation are promising directions.

## Figures and Tables

**Table 1:** Composition of the multivariate input used for PV power forecasting.

Category	Variables	Description	Unit
NWP predictors	nwp_globalirrad	Forecasted global irradiance	W/$m^{2}$
	nwp_directirrad	Forecasted direct irradiance	W/$m^{2}$
	nwp_temperature	Forecasted ambient temperature	∘C
	nwp_humidity	Forecasted relative humidity	%
	nwp_windspeed	Forecasted wind speed	m/s
	nwp_winddirection	Forecasted wind direction	∘
	nwp_pressure	Forecasted atmospheric pressure	hPa
On-site observations	lmd_totalirrad	Measured total irradiance	W/$m^{2}$
	lmd_diffuseirrad	Measured diffuse irradiance	W/$m^{2}$
	lmd_temperature	Measured ambient temperature	∘C
	lmd_pressure	Measured atmospheric pressure	hPa
	lmd_winddirection	Measured wind direction	∘
	lmd_windspeed	Measured wind speed	m/s
Historical PV output	power	Actual PV power, used as an autoregressive input	MW

**Table 2:** Performance comparison of forecasting modes using different input features.

Method	RMSE	MAE	Pearson *r*	CR (%)
Historical power	0.0414	0.0239	0.976	95.8
Raw NWP	0.0328	0.0194	0.985	96.7
Downscaled NWP	0.0184	0.0112	0.995	98.1

**Table 3:** Performance comparison of forecasting model architectures.

Method	RMSE	MAE	Pearson *r*	CR (%)
LSTM	0.0577	0.0321	0.953	94.2
CNN-LSTM	0.0426	0.0247	0.974	95.7
AC-LSTM	0.0181	0.0111	0.995	98.1

## Data Availability

The original contributions presented in this study are included in the article. Further inquiries can be directed to the corresponding author.
